# Assessment of MALDI-TOF MS biotyping for *Borrelia burgdorferi* sl detection in *Ixodes ricinus*

**DOI:** 10.1371/journal.pone.0185430

**Published:** 2017-09-26

**Authors:** Pierre H. Boyer, Nathalie Boulanger, Amira Nebbak, Elodie Collin, Benoit Jaulhac, Lionel Almeras

**Affiliations:** 1 Early Bacterial Virulence: Lyme borreliosis Group, Université de Strasbourg, Fédération de Médecine Translationnelle de Strasbourg, VBP EA 7290, Strasbourg, France; 2 French National Reference Center for *Borrelia*, Hôpitaux Universitaires de Strasbourg, Strasbourg, France; 3 Aix Marseille Université, Unité de Recherche en Maladies Infectieuses et Tropicales Emergentes (URMITE), UM63, CNRS 7278, IRD 198 (Dakar, Sénégal), Inserm 1095, Institut Hospitalo-Universitaire Méditerranée Infection 19–21 Boulevard Jean Moulin, Marseille, France; 4 Laboratoire de Biodiversité et Environnement: Interactions génomes, Faculté des Sciences Biologiques, Université des Sciences et de la Technologie Houari Boumediene (USTHB), Bab Ezzouar, Algiers, Algeria; 5 Unité de Parasitologie et Entomologie, Département des Maladies Infectieuses, Institut de Recherche Biomédicale des Armées, Marseille, France; University of Maryland, College Park, UNITED STATES

## Abstract

Matrix Assisted Laser Desorption/Ionization Time-of-Flight Mass Spectrometry (MALDI-TOF MS) has been demonstrated to be useful for tick identification at the species level. More recently, this tool has been successfully applied for the detection of bacterial pathogens directly in tick vectors. The present work has assessed the detection of *Borrelia burgdorferi* sensu lato in *Ixodes ricinus* tick vector by MALDI-TOF MS. To this aim, experimental infection model of *I*. *ricinus* ticks by *B*. *afzelii* was carried out and specimens collected in the field were also included in the study. *Borrelia* infectious status of *I*. *ricinus* ticks was molecularly controlled using half-idiosome to classify specimens. Among the 39 ticks engorged on infected mice, 14 were confirmed to be infected by *B*. *afzelii*. For field collection, 14.8% (n = 12/81) *I*. *ricinus* ticks were validated molecularly as infected by *B*. *burgdorferi sl*. To determine the body part allowing the detection of MS protein profile changes between non-infected and *B*. *afzelii* infected specimens, ticks were dissected in three compartments (*i*.*e*. 4 legs, capitulum and half-idiosome) prior to MS analysis. Highly reproducible MS spectra were obtained for *I*. *ricinus* ticks according to the compartment tested and their infectious status. However, no MS profile change was found when paired body part comparison between non-infected and *B*. *afzelii* infected specimens was made. Statistical analyses did not succeed to discover, per body part, specific MS peaks distinguishing *Borrelia*-infected from non-infected ticks whatever their origins, laboratory reared or field collected. Despite the unsuccessful of MALDI-TOF MS to classify tick specimens according to their *B*. *afzelii* infectious status, this proteomic tool remains a promising method for rapid, economic and accurate identification of tick species. Moreover, the singularity of MS spectra between legs and half-idiosome of *I*. *ricinus* could be used to reinforce this proteomic identification by submission of both these compartments to MS.

## Introduction

Lyme borreliosis is the most prevalent vector borne disease in the northern hemisphere [1]. This multi-systemic disease presents a large variety of associated clinical signs which hamper clinical diagnosis. Lyme disease is caused by bacteria belonging to the species complex *Borrelia burgdorferi* sensu lato (sl). *B*. *burgdorferi* sl complex includes, up to now, 21 known bacteria species [2]. Among these species 5 are commonly found in human pathology, and *B*. *afzelii* is the most prevalent bacterium in Europe [1]. These pathogens are transmitted by blood feeding of infectious ticks belonging to the *Ixodes* genus. In western Europe, *I*. *ricinus* is the most common vector [1].

Until now, there is no human licensed vaccine available, prevention and vector controls remain the main protective measures. To inform populations and to establish efficient protective devices, realization of vector epidemiological studies is required to map tick species and to determine their *Borrelia* infectious status. Tick species identification could be carried out morphologically and the detection of *B*. *burgdorferi* sl. is mainly achieved using molecular biology methods [3]. The expertise required for tick morphological identification and the high costs and time necessary for molecular pathogen detection are limitation factors for rapid characterization of tick species and their *Borrelia* infectious status.

Recently, Matrix Assisted Laser Desorption/Ionization Time-of-Flight Mass Spectrometry (MALDI-TOF MS) has been successfully applied for the identification of several arthropod families, including ticks [4]. Different tick body parts could be used for their identification by MS, either using whole specimens [5] or tick legs [6,7]. As protein repertory is not equivalent according to tick body part of the same species, the same tick compartment should be used for MS spectra queried against the MS reference database and database creation.

Moreover, this last decade, MALDI-TOF MS has been introduced in clinical microbiology laboratories for the identification and classification of micro-organisms, including bacteria and yeast [8]. The sample preparation simplicity, rapidity and reagent low-costs contributed to the popularity of this tool for microbial routine analyses [9]. Consequently, based on the success of this tool for bacteria and tick identification, MALDI-TOF MS was assessed for the determination of tick bacteria-infectious status. A pioneering study established the proof-of-concept of *Borrelia crocidurae* detection in soft ticks, *Ornithodoros sonrai*, by submitting leg protein extracts to MS [10]. More recent works, studying the detection of *Rickettsia* spp. pathogens in infected ticks using MALDI-TOF MS, reported the dual identification of tick species and *Rickettsia* spp. infectious status using tick legs [11] or tick hemolymph [12]. Indeed, reproducible changes in MS protein profiles were observed between *Rickettsia*-free and–infected conspecific specimens.

The aim of the present study was to assess MALDI-TOF MS ability to detect *B*. *afzelii* in *I*. *ricinus* tick vector which could be useful for entomological diagnosis. *I*. *ricinus* ticks collected in the field or infected experimentally by *B*. *afzelii* NE4049 were used for this demonstration. The development of a rapid, economic and reliable method for dual identification; the determination of tick species and their *Borrelia* infectious status, is becoming more and more essential in the framework of Lyme disease vectors monitoring and pathogen circulation.

## Material and methods

### *Borrelia afzelii* NE4049 culture

*Borrelia afzelii* NE4049 [13] was cultured in the BSK-H medium (Sigma, Saint Quentin Fallavier, France) under anaerobic conditions for 8 days. The medium was then centrifuged and the pellet washed three times with PBS. Finally, the pellet was resuspended with 100 μL of PBS and borrelial density (bacteria/μL) was determined using a Petroff-Hausser counting chamber.

### Ticks

A total of 149 *I*. *ricinus* ticks collected in the field (n = 81) or laboratory-reared (n = 68) were used for this study. Field collection of ticks was done in the Murbach area (GPS: N47.918961 / E007.210436; Alsace, France) in June 2016, by dragging a white flannel flag (1x1 m) over low vegetation. Ticks species were determined by morphological identification under a binocular loupe at a magnification of ×56 (Leica M80, Leica, Nanterre, France) using taxonomic keys [14]. Only *I*. *ricinus* ticks were included in this work. The laboratory rearing of *I*. *ricinus* ticks was performed as previously described [15,16]. For *Borrelia* experimental infections of ticks, *I*. *ricinus* specimens from the larva stage were fed on C3H/HeN mice infected (n = 39) by *B*. *afzelii* strain NE4049 or not infected (n = 29) as previously described [16,17]. At the nymphal stage, tick specimens were frozenly killed until future analyses.

Mice used in tick experiments were three to four-week-old C3H/HeN pathogen-free. They were obtained from our breeding colony and provided food and water *ad libitum*. At the end of the experiment, mice were killed by isoflurane gas overdose.

### Tick dissection

Ticks were processed as previously described [6]. Briefly, each specimen was rinsed 1 minute once with 70% (v/v) ethanol, twice with distilled water and then air-dried. Ticks were individually dissected with a sterile surgical blade under a binocular loupe. Four legs and the capitulum were removed and the idiosome was longitudinally cut in two equal parts. The half-idiosome harboring legs were used for molecular analysis. The three other body parts, the four legs, the capitulum and the half-idiosome without the legs, were prepared individually for MALDI-TOF MS analysis. For ticks collected in the field, only the four legs and the half-idiosome legs less, were submitted to MALDI-TOF MS, the remaining body part was used for molecular analyses.

### DNA extraction

DNA of each half-idiosome with legs was individually extracted with ammonium hydroxide (Sigma-Aldrich) as previously described [18,19]. Purified DNA from each tick specimen was either immediately used or stored at -80°C until use.

### Molecular identification of ticks

DNA from 14 morphologically identified *I*. *ricinus* specimen collected in the field were validated by sequencing a PCR fragment of 480 bp from cytochrome oxidase I (COI) gene as previously described [20]. The sequences were analyzed using the 4Peaks software (version 1.7.1) (Softnic^®^ Corporate, Barcelona, Spain), and were then blasted against GenBank (http://blast.ncbi.nlm.nih.gov).

### Molecular detection of *B*. *burgdorferi* sl in ticks

The presence of *B*. *burgdorferi* sl in ticks was determined by a real-time PCR assay targeting a conserved region of the flagellin gene. Species genotyping was achieved by melting curve analysis as previously described [21]. DNA from *B*. *afzelii*, *B garinii* and *B*. *burgdorferi* ss were added or not to the PCR mix as positive and negative controls, respectively.

### Sample homogenization and MALDI-TOF MS analysis

Each compartment dissected was homogenized individually using a FastPrep-24 device (MP Biomedicals, Illkirch-Graffenstaden, France) and glass beads (Sigma, Lyon, France) in a mix (50/50) of 15 μL 70% (v/v) formic acid (Sigma) and 15 μL 50% (v/v) acetonitrile (Fluka, Buchs, Switzerland) for protein extraction according to the standardized automated setting described by Nebbak *et al*. [22]. A quick spin centrifugation at 200 g for 1 min was then performed and 1 μL of the supernatant of each sample was spotted on the MALDI-TOF steel target plate in quadruplicate (Bruker Daltonics, Wissembourg, France). After air-drying, 1 μL of matrix solution composed of saturated α-cyano-4-hydroxycinnamic acid (Sigma, Lyon, France), 50% (v/v) acetonitrile, 2.5% (v/v) trifluoroacetic acid (Aldrich, Dorset, UK) and HPLC-grade water was added. Matrix solution was loaded in duplicate onto each MALDI-TOF plate with and without bacterial control (*Pseudomonas aeruginosa* ATCC 27853) respectively as a positive or negative control. Spectra were acquired on a Microflex LT MALDI-TOF Mass Spectrometer (Bruker Daltonics) as previously described [23].

### Mixing *Borrelia* with tick protein extracts

Pelleted *B*. *afzelii* were suspended in water to obtain 1.10^7^ bacteria/μL. Serial dilution in water were done to add 10^6^, 10^5^ or 10^4^ bacteria to half-idiosome protein extract from *Borrelia-*free *I*. *ricinus*. One microliter of this mix was spotted in quadruplicate on the MALDI-TOF target plate. Half-idiosome protein extract from *Borrelia-*free *I*. *ricinus* and *B*. *afzelii* bacteria protein extract were loaded on the MS target plate as controls.

### MS spectra analysis

MS spectra profiles were firstly controlled visually with flexAnalysis v3.3 software (Bruker Daltonics). MS spectra were then exported to ClinProTools v2.2 and MALDI-Biotyper v3.0. (Bruker Daltonics) for data processing (smoothing, baseline subtraction, peak picking). MS spectra reproducibility was assessed by the comparison of the average spectral profiles (MSP, Main Spectrum Profile) obtained from the four spots for each specimen according to body part and infectious status with MALDI-Biotyper v3.0 software (Bruker Daltonics). MS spectra reproducibility and specificity taking into account tick body part and *Borrelia* infectious status were objectified using clustering analyses and Composite Correlation Index (CCI) tool. Cluster analyses (MS dendrogram) were performed based on comparison of the MSP given by MALDI-Biotyper v3.0. software and clustered according to protein mass profile (i.e., their mass signals and intensities). The CCI tool from MALDI-Biotyper v3.0. software was also used, to assess the spectral variations within and between each sample group, according to the body part and *Borrelia* infectious status, as previously described [23,24]. Higher correlation values (expressed by mean ± standard deviation–SD) reflect higher reproducibility for the MS spectra, and were used to estimate MS spectra distance for each condition (body part and *Borrelia-*infectious status). To visualize MS spectra distribution from ticks collected in the field, according to body part and/or *Borrelia*-infectious status, principal component analysis (PCA) from ClinProTools v2.2 software was performed. To list discriminating peaks between compartments and/or infectious status, MS spectra were analysed using the genetic algorithm (GA) model from ClinProTools v 2.2 software. The maximum number of generations was set to 250 and the number of neighbours was three for K nearest neighbour (KNN) classification. A manual inspection and validation of the selected peaks gave recognition capability (RC) value together with the highest cross-validation (CV) value to assess the reliability and accuracy of the model. The discriminating peak masses generated by the model were searched in the peak report created for each compartment and condition.

### Database creation and blind tests

The reference MS spectra were created using spectra from legs and half-idiosomes of five *I*. *ricinus* ticks at the nymphal stage using MALDI-Biotyper software v3.0. (Bruker Daltonics) [25]. MS spectra were created with an unbiased algorithm using information on the peak position, intensity and frequency. The reproducibility of the MS profiles per body part was evaluated with MALDI-Biotyper software v3.0., which assigns log score values (LSVs) based on the degree of confidence with which the query spectrum identifies to the reference spectrum. LSVs ranged from 0 to 3. According to previous studies [6,23], a LSV of at least 1.8 should be obtained to be considered reliable for species identification. Data were analysed by using GraphPad Prism software version 5.01 (GraphPad, San Diego, CA, USA).

### Ethical statement

The protocols to maintain tick colony (N°APAFIS 886–2015062209279407) and to infect ticks on *Borrelia* infected mice (N°APAFIS 885–2015062209113374) were approved by the Comité Régional d’Ethique en Matière d’Expérimentation Animale de Strasbourg (CREMEAS—Committee on the Ethics of Animal Experiments of the University of Strasbourg). These protocols follow the European directive 2010/63/EU. The authority who issued the permission to collect ticks for each location was the ONF (Office National des forêts, France). The field studies did not involve endangered or protected species.

## Results and discussion

### Molecular detection of *B*. *burgdorferi* sl in ticks

Among the 39 *I*. *ricinus* larva, laboratory-reared and experimentally exposed to mice infected by *B*. *afzelii*, 36% of the ticks (14/39) were found infected by *B*. *afzelii* at the nymphal stage, according to real-time PCR results. The absence of *B*. *afzelii* was confirmed by RT-PCR in the 29 *I*. *ricinus* nymphs, laboratory-reared and engorged on pathogen-free mice at the larva stage.

A total of 81 ticks at the nymphal stage were collected by flagging in the Murbach area (France). All these specimens were classified as *I*. *ricinus* by morphological identification. Among them, the DNA extracted from half-idiosome of 13 specimens, randomly selected, were submitted to tick species identification by molecular tool. BLAST analyses corroborated morphological identification showing 99% sequence similarities with *I*. *ricinus* COI sequence from GenBank (Accession number: KF197134.1). Concerning their *Borrelia* infectious status, 12 ticks (14.8%) were found infected, including six, three and two specimens by *B*. *afzelii*, *B*. *burgdorferi* ss and *B*. *garinii*, respectively, and one co-infected by *B*. *garinii* and *B*. *burgdorferi* ss. This rate of infection is commonly found in this area [3] and *B*. *afzelii* is the most prevalent species in Europe [26].

### Assessment of MS spectra specificity between *Borrelia*-free and–infected ticks according to body part

The research of MS spectra changes according to *I*. *ricinus* infectious status was done in three body compartments, the four legs, the capitulum and the half-idiosome legs less. The legs were chosen because all anterior descriptions of distinctive MS spectra in ticks between pathogen-free and bacteria-infected specimens were achieved using legs [10,11] or hemolymph collected at the legs level [12]. The detection of *B*. *crocidurae* in the legs of soft ticks and some *Rickettsia* species in the legs and hemolymph of hard ticks underlined that these bacteria disseminate in the ticks at sufficiently elevated concentration to modify MS spectra. The capitulum was tested to comfort the specificity of leg MS profile changes following *Borrelia* infection. Indeed, if MS profile changes were detected at the leg level, it appeared essential to submit to MS another body part of the same tick specimen to assess whether MS pattern modifications were also detected in this second compartment and whether some of these MS peak changes were shared between legs and capitulum. Finally, as the presence of *B*. *burgdorferi* sl was repeatedly reported in the gut of infected *I*. *ricinus* ticks [27,28], the half-idiosomes were also submitted to MS analysis.

#### Reproducibility of MS spectra according to tick compartment

**Fig 1:**
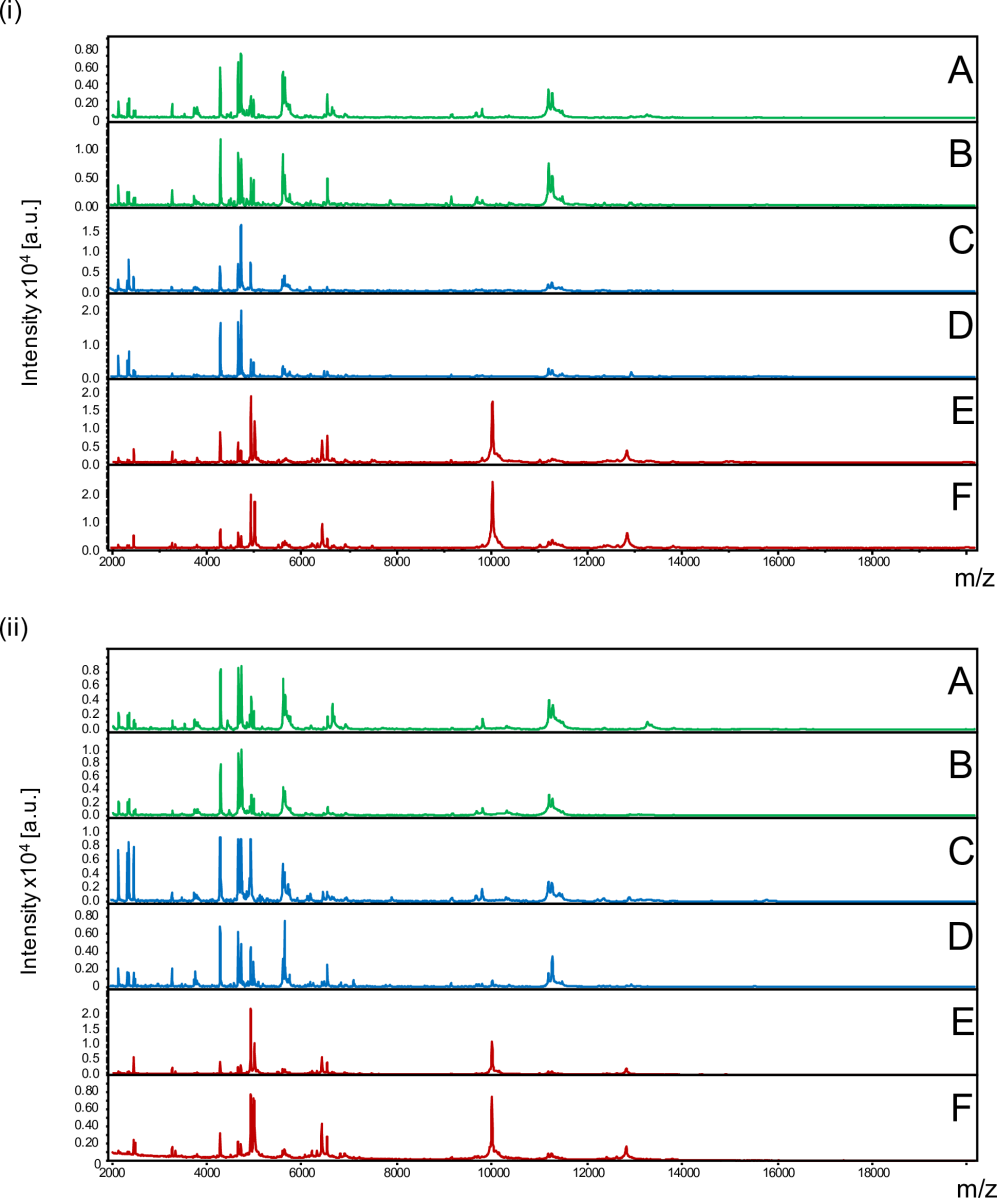
Comparison of MALDI-TOF MS spectra from legs, capitula and half-idiosomes of adult *I*. *ricinus* pathogen-free (i) or infected by *Borrelia afzelii* (ii). Representative MS spectra of legs (A, B), capitula (C, D) and half-idiosomes (E, F) from laboratory reared *I*. *ricinus* homogenized automatically using FastPrep-24 device with glass powder. a.u., arbitrary units; m/z, mass-to-charge ratio.

Before comparing MS spectra from the same tick compartment according to the *Borrelia*-infectious status, reproducible MS spectra had to be obtained for *I*. *ricinus* for each compartment and *Borrelia*-infectious status. The MS spectra comparison from five laboratory reared specimens infected (Fig 1 –ii) or not (Fig 1 –i) by *Borrelia* visually revealed reproducible protein profiles for each body part. Interestingly, MS spectra from legs (A,B) and capitula (C,D) were closely related. Conversely, MS spectra from half-idiosomes (E,F) appeared more distinct compared to the MS spectra from the two other body parts. Nevertheless, for each body part, no visible MS peak clearly distinguished *Borrelia-*infected (ii) from pathogen-free specimens (i).

#### Specificity of MS spectra according to tick Borrelia-infectious status

**Fig 2:**
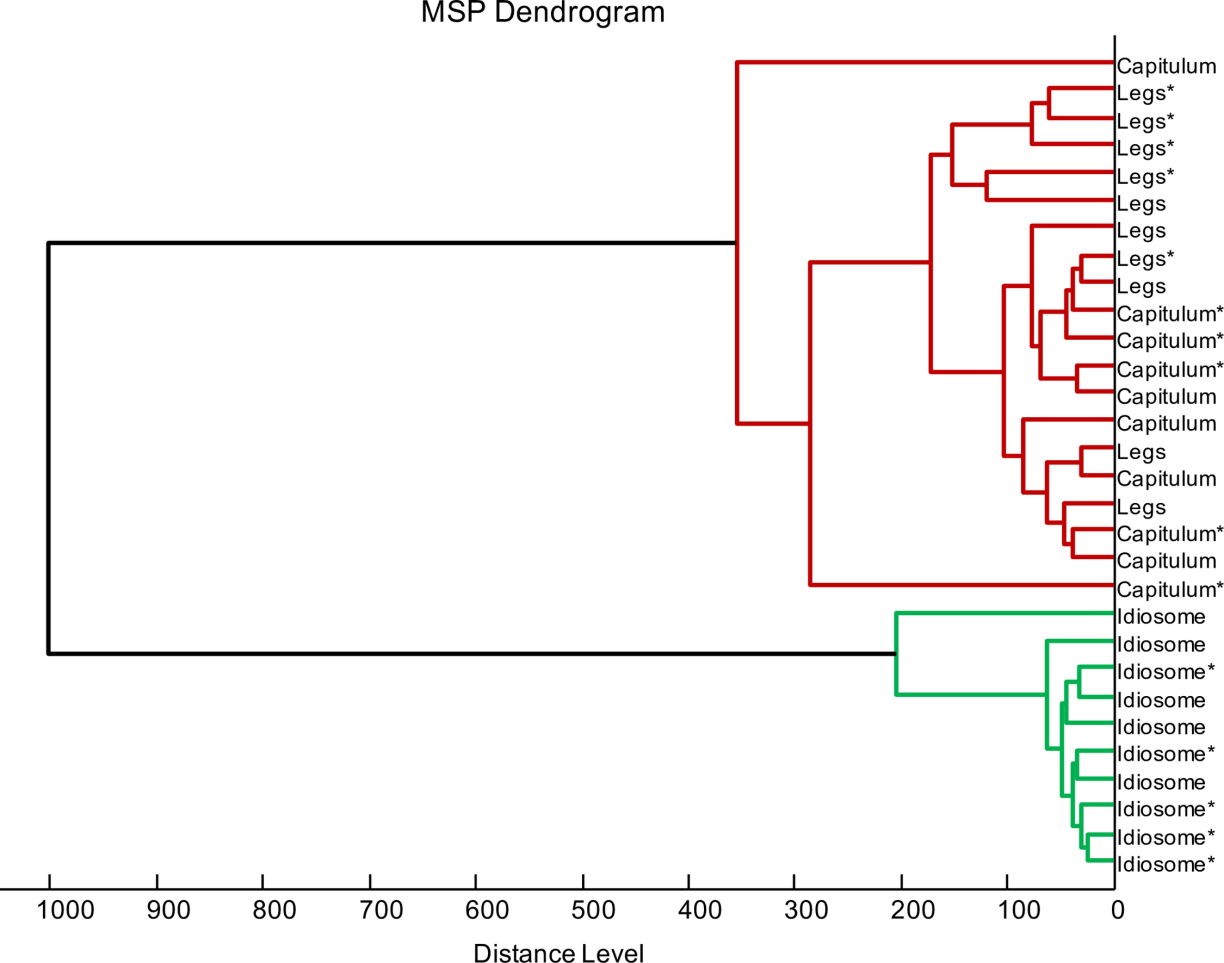
MSP dendrogram of MALDI-TOF MS spectra from legs, capitula and half-idiosomes of adult *I*. *ricinus* pathogen-free or infected by *Borrelia afzelii*. Five specimens per body part and *B*. *afzelii* infectious status were used to construct the dendrogram. The dendrogram was created using Biotyper v3.0 software and distance units correspond to the relative similarity of MS spectra. The specimens infected by *B*. *afzelii* were indicated by asterisks (*).

**Fig 3:**
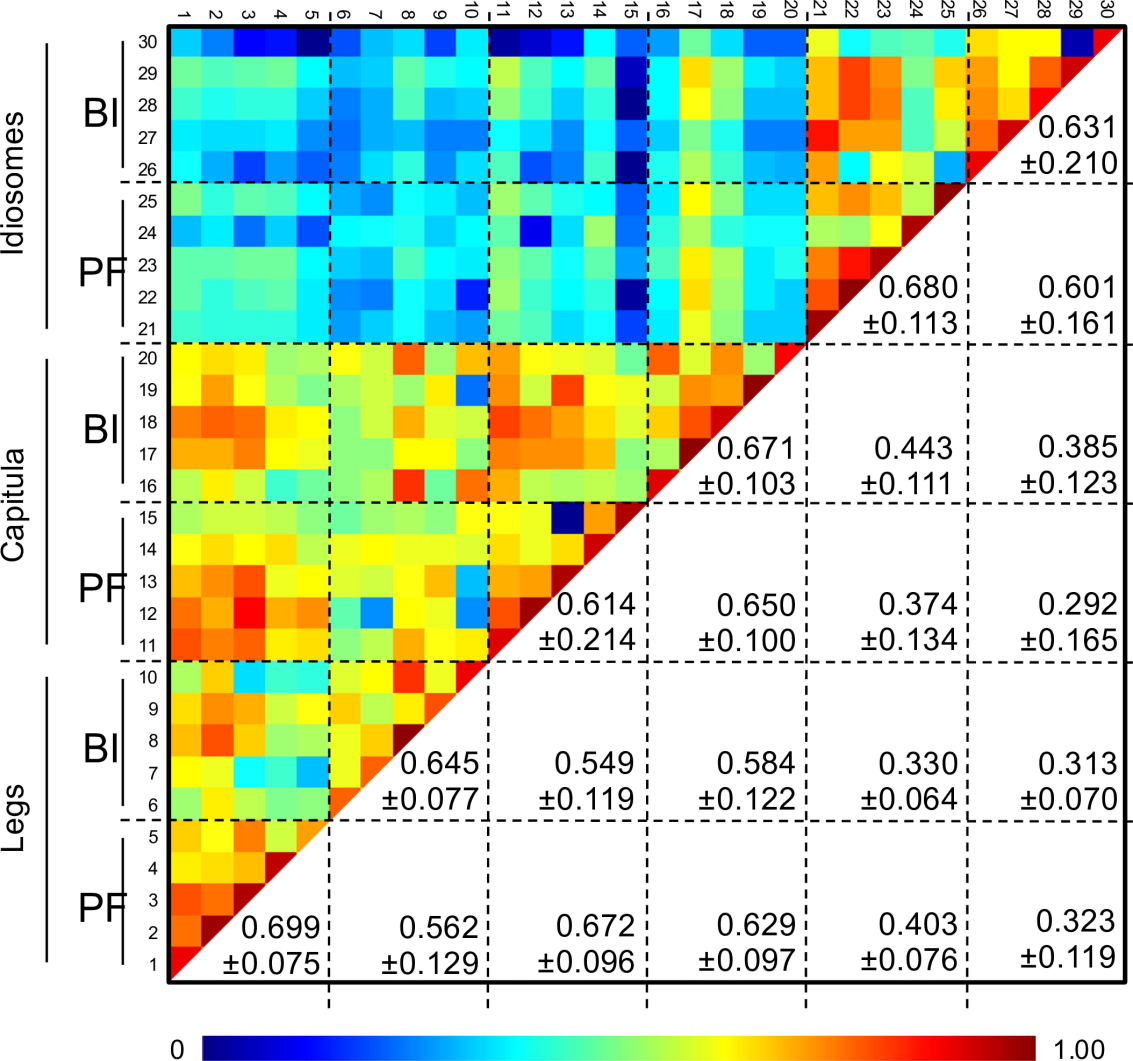
Assessment of *I*. *ricinus* MS spectra reproducibility according to tick body parts and *Borrelia* infectious status using composite correlation index (CCI). MS spectra from five specimens per body part and *B*. *afzelii* infectious status were analysed using the CCI tool. Body part and infectious status are indicated on the left side of the heat map. Levels of MS spectra reproducibility are indicated in red and blue revealing relatedness and incongruence between spectra, respectively. CCI matrix was calculated using MALDI-Biotyper v3.0. software with default settings (mass range 3.0–12.0 kDa; resolution 4; 8 intervals; auto-correction off). The values correspond to the mean coefficient correlation and respective standard deviations obtained for paired condition comparisons. CCI were expressed as mean ± standard deviation. BI, *Borrelia*-infected; PF, pathogen-free.

To assess whether specific MS profiles could be associated to tick infectious status per body part, five MS profiles per condition were used to perform clustering and correlation analyses. The dendrogram showed that half-idiosome MS spectra clustered on distinct branches from legs and capitula (Fig 2). Moreover, these last two body parts were found imbricated suggesting the absence of specific MS profiles distinguishing legs and capitula. Additionally, an overlapping of MS spectra from *Borrelia-*infected and pathogen-free specimens was observed for each compartment on the dendrogram. These results indicated that MS protein profiles were weakly affected by *Borrelia* infection and comforted the low specificity of MS spectra to distinguish tick legs and capitula. These data were confirmed by CCI matrix highlighting a low correlation of MS spectra between idiosomes and legs or capitula (mean±SD: 0.358 ± 0.121; Fig 3). The high CCI obtained between MS spectra from legs and capitula regardless of their infectious status also strongly suggest the lack of specificity in the MS profile. The superimposition of average MS profiles from idiosomes between *Borrelia-*infected and pathogen-free specimens using ClinProTool software (Bruker), did not reveal differences in peak position. The detection of few peaks of low intensity modifications could explain the decrease of MS spectra correlation for paired comparison of the idiosome profiles according to *Borrelia*-infectious status. These intensity MS peak changes could be attributed to tick response to bacterial infection. Indeed, previous works reported transcriptome [29] or protein repertoire [30] changes according to the infection tick status. However, the MS profile changes appeared insufficient to associate a specific protein pattern to *I*. *ricinus* infected by *Borrelia*. The appearance and/or disappearance of at least a few MS peaks following tick infection by a specific *Borrelia* seems necessary to sustain the detection of a specific MS profile.

### Comparison of I. ricinus MS profiles between laboratory-reared and field collected specimens

**Fig 4:**
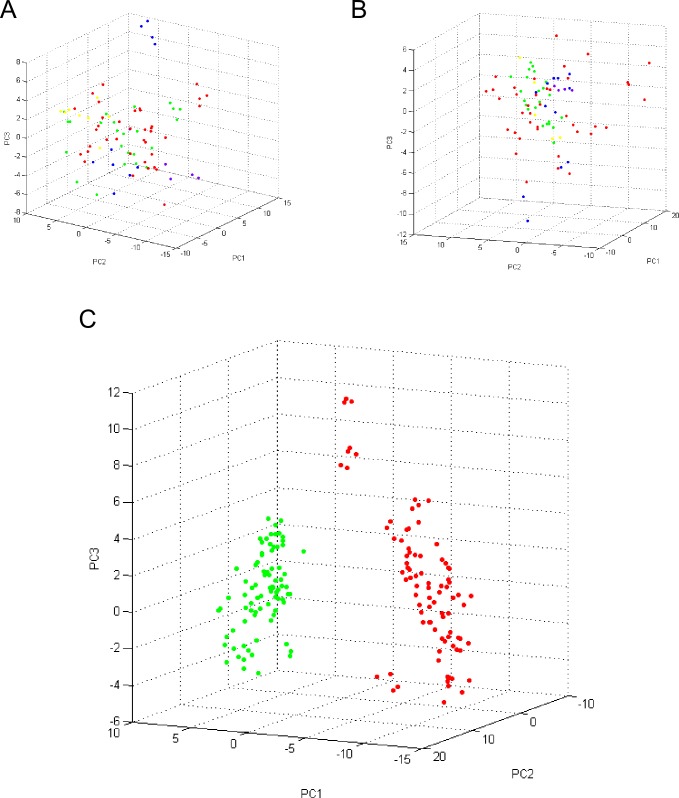
Principal component analysis (PCA) from MS spectra of idiosomes and legs of *I*. *ricinus* infected or not by *Borrelia sp*. PCA dimensional image from MS spectra of *I*. *ricinus* idiosomes (A) and legs (B) *Borrelia*-free (red dots, n = 10), infected by *B*. *afzelii* (green dots, n = 6), *B*. *burgdorferi* (blue dots, n = 3), *B*. *garinii* (yellow dots, n = 2), co-infected by *B*. *garinii* and *B*. *burgdorferi* (purple dots, n = 1). (C) PCA dimensional image from the same MS spectra of *I*. *ricinus* idiosomes (red dots, n = 22) and legs (green dots, n = 22). The contributions of PC1, PC2 and PC3 were 38.4%, 15.5% and 7.2%, respectively. Among the *Borrelia*-free specimens, five were laboratory reared and the other five came from field collection.

A comparison of MS spectra distribution from specimens collected in the field taking into account diversity of *Borrelia* species identified in *I*. *ricinus* ticks was performed. To visualize their distribution according to their *Borrelia*-infectious status, PCA was performed. Twelve *I*. *ricinus* ticks (co)-infected by a *B*. *burgdorferi* sl species, plus five *Borrelia*-free specimens and five laboratory reared *I*. *ricinus* ticks were included in this analysis. An intertwining of the dot-reflecting MS spectra distribution from half-idiosomes (Fig 4A) and legs (Fig 4B) was observed independently of their infectious status. No clustering was found for MS spectra from ticks infected neither by the same *Borrelia* species, nor between *Borrelia*-free and–infected specimens in each compartment (Fig 4A and 4B). Then, these results strengthened the uselessness of MALDI-TOF MS to distinct *I*. *ricinus* specimens according to their *Borrelia*-infectious status in both these body parts. Interestingly, the dots corresponding to MS spectra from *Borrelia*-free specimen, laboratory reared and collected in the field, overlapped, underlining the specificity of the protein profiles to each *I*. *ricinus* body part independently of their origins (*i*.*e*., laboratory or field).

Conversely, the submission of these same last 22 samples per body part to PCA highlighted a clear separation of the dots from the legs and idiosomes (Fig 4C), confirming a specificity of MS spectra between these two compartments. Thirteen discriminating MS peaks were exhibited using the Genetic Algorithm model (ClinProTools software) between idiosomes and legs from five laboratory reared and pathogen-free *I*. *ricinus* specimens (Table 1). Recognition capability (RC) and a cross validation (CV) 100% values were obtained which confirms their accuracy for tick identification. Indeed, MS profiles are poorly affected by the environmental conditions [31] and, even specific, there is a cross recognition between the *Rickettsia*-free and infected tick [11].

**Table 1:** Mass peak list distinguishing legs and idiosomes from laboratory reared and pathogen-free *I*. *ricinus* specimens.

Mass (m/z)	Start Mass	End Mass	Legs	Idiosome
3329.42	3321.25	3335.84	-	+
3724.17	3716.85	3728.83	+	-
3751.09	3742.66	3755.75	+	-
4453.72	4446.31	4463.82	+	-
4721.00	4717.60	4727.42	+	-
4996.13	4981.44	5005.29	-	+
5496.18	5483.74	5503.89	-	+
5701.28	5695.40	5716.65	+	-
6203.19	6188.93	6213.30	-	+
6311.90	6293.38	6321.68	-	+
6411.56	6395.70	6421.23	-	+
9991.35	9975.31	10009.66	-	+
12821.56	12769.80	12859.35	-	+
Total			5	8

Da. Daltons; m/z. mass to charge.

The inefficiency of MS spectra to distinguish *Borrelia*-infected from pathogen-free *I*. *ricinus* ticks regardless of the body part tested could be attributed to different factors. Firstly, at a distance of blood feeding, *B*. *burdorgferi* bacteria are mainly confined to tick gut [27]. The sequestration of the *Borrelia* in the gut could explain the lack of probative MS spectra changes in the legs and capitula of *B*. *afzelii* infected ticks. Moreover, the bacterial load seems to be decreasing over time especially for laboratory-reared ticks [32]. On the contrary, *R*. *slovaca*, *R*. *conorii* and *R*. *massilliae* can be observed directly by tick hemolymph drop staining [33]. The dissemination of bacteria to the entire tick body vectors explains their detection in legs [11] and hemolymph of ticks [12].

The inability to distinguish *Borrelia*-infected from pathogen-free *I*. *ricinus* ticks at the idiosome level could be attributed, on the one hand, to the low gut *Borrelia* inoculum in unfed ticks (approximately 2000 spirochetes [27]) and, on the other hand, to the variety of other resident bacteria species hiding their detection [34]. The blood meal was reported to ensure the gut multiplication and the dissemination of the *Borrelia* to the tick salivary glands [27]. Then, the research of MS profiles changes in ticks recently blood-engorged could be an alternative. Nevertheless, the blood contained in the idiosomes from freshly engorged ticks was already reported to strongly modify MS spectra, impairing tick identification [5].

An alternative proteomic strategy for elucidation tick *B*. *burgdorferi* sl*-*infectious status could be to analyze peptide MS profiles instead of intact proteins. This approach was successfully applied for *Culicoides* species identification [35]. With this method, it would be possible to identify unambiguously differential MS peaks attributed to *B*. *burgdorferi* sl*-*infection by peptide sequencing using tandem MS (MS/MS). However, the resort to more sophisticated mass spectrometry apparatus is required such as MALDI-TOF MS/MS or liquid chromatography electrospray ionization (LC/ESI) MS/MS machines [35]. The MS window range was also change (i.e., 400 to 4kDa), hampering the use of resulting MS spectra to query the home-made MS reference database for tick species identification done on MALDI-TOF MS. Moreover, an additional step consisting in sample enzymatic digestion (e.g. Trypsin) prior to MS submission is necessary. The combination of the sample preparation steps plus tandem MS analysis not only increases dramatically the processing time for determination of tick infectious status from few minutes by MALDI-TOF MS [4,10] to several hours, but also the cost per analyze. Taken together, tandem MS could appear less attractive compared to molecular biology for tick *Borrelia burgdorferi sl-*infectious status. Nevertheless, proteomic methods present the advantage to detect pathogenic agent protein products which is more convincing to claim that bacteria are alive than DNA amplification which could correspond to trace of death bacteria. It also participates in the demonstration of vector competence of an arthropod vector [36].

#### MS profile change following the addition of B. afzelii to half-idiosome

**Fig 5:**
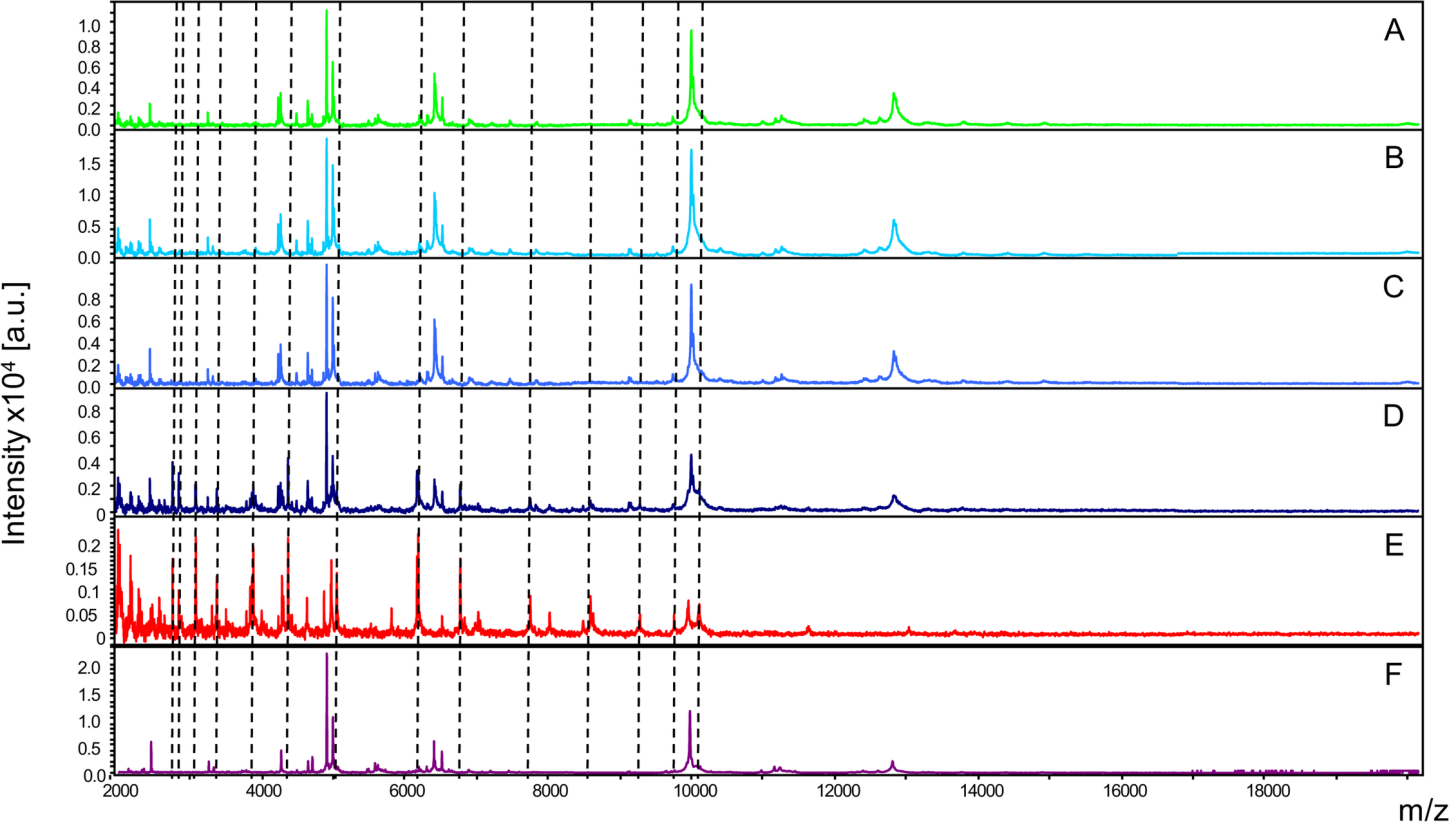
Sensitivity of MALDI-TOF MS for *Borrelia* detection in mix protein extract. Representative MS spectra from half-‡idiosome *I*. *ricinus* protein extract, without (A) or with the addition of 10^4^ (B), 10^5^ (C) or 10^6^ (D) *Borrelia afzelii* bacteria. MS profiles from 10^6^ *Borrelia afzelii* alone (E) and half-idiosome protein extract from *I*. *ricinus* infected by *Borrelia afzelii* (F) were shown. MS peaks commonly found between *B*. *afzelii* and half-idiosome *I*. *ricinus* protein extract with the addition of 10^6^ were indicated by dashed lines.

The ratio of bacteria/tick protein abundances seems then too low for tick species and *Borrelia* detection by MS. To evaluate sensitivity of MALDI-TOF MS for this dual detection, serial dilutions of *B*. *afzelii* were added to half-idiosome *I*. *ricinus Borrelia*-free protein extract. The comparison of the resulting MS profiles of these mix samples with unmixed ones, revealed that MS peaks were shared with *B*. *afzelii* MS spectra, only for the tick sample added with the highest bacteria concentration (Fig 5). Interestingly, MS peaks were neither commonly found between sample mix of tick and half-idiosome with *Borrelia*-infected *I*. *ricinus* specimens. The MS spirochetes detection limit appeared to be around 10^6^ *Borrelia* per half-idiosome, which is 500 fold upper than the borrelial inoculum found in a *Borrelia*-infected unengorged nymph [27]. Conversely, our molecular assay detects *B*. *burgdorferi* sl DNA to a concentration of 2 bacteria/μL [37].

### MS reference spectra database creation and validation step

The MS spectra from legs, capitula and idiosomes of the 5 specimens, laboratory-reared and non-exposed, used for clustering analysis, were loaded into MALDI-Biotyper v.3.0. (Bruker Daltonics) to create a reference MS database. Then, the remaining 144 legs, 144 idiosomes and 63 capitula of *I*. *ricinus* laboratory reared or collected in the field, infected or not by *Borrelia*, were subjected to MALDI-TOF MS analysis. Overall, 98.9% (347/351) of the MS spectra queried against the database, obtained LSVs over 1.8, the threshold established for relevant identification [4]. The four samples which did not reach the LSV threshold were attributed to low quality of MS spectra. The implementation of a preprocessing step of quality control should be developed to remove low quality MS spectra which may induce irrelevant identification [38].

**Fig 6:**
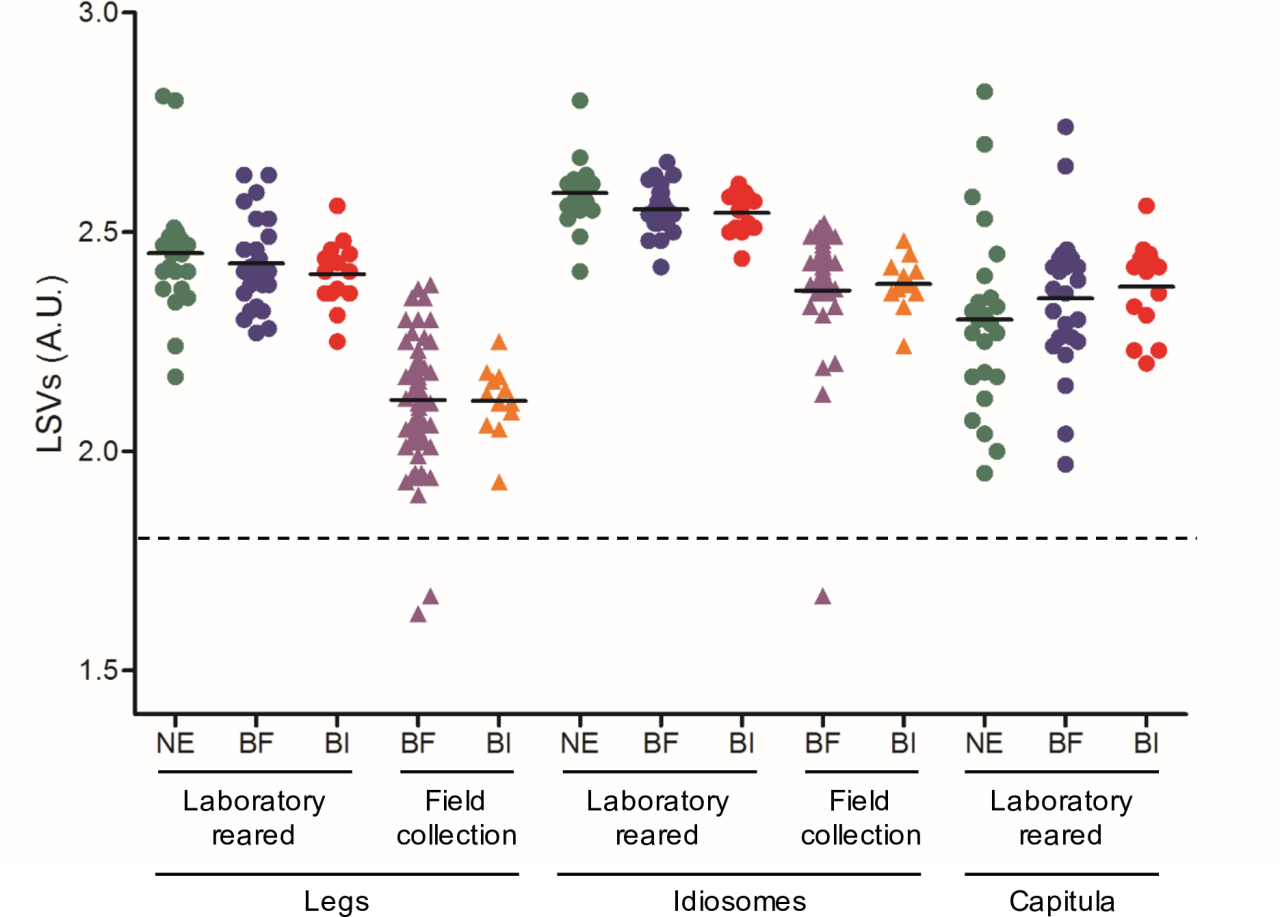
Comparison of LSVs from MS spectra of *I*. *ricinus* ticks according to body part, origin and *Borrelia*-infectious status. Dashed line represent the threshold value for relevant identification (LSVs>1.8). LSV, log score value; NE, non-exposed; BF; *Borrelia*-free; BI; *Borrelia*-infeted.

Among MS spectra with LSVs over 1.8, 100%, 97.2% and 73.0% of the idiosome, leg and capitula MS spectra, respectively, yielded correct identification of *I*. *ricinus* body part. For legs and capitula, cross-recognition occurred due to the proximity of the MS spectra between these two body parts, as reported above. However, for laboratory reared specimens, lower heterogeneity of LSVs from legs and idiosomes were obtained in comparison to capitula (Fig 6).

Interestingly, LSV ranges were equivalent between *Borrelia*-infected and–free, for each body part, comforting the absence of MS profile changes according to *Borrelia*-infectious status (Fig 5).

The MS spectra specificity of legs and idiosomes from *I*. *ricinus* allows to submit these two body parts independently to MS for specimen identification. The corroboration of the species determination using two distinct body parts from the same specimen should reinforce identification by this proteomic tool. In the future, the creation of MS spectra reference database using both these compartments could improve specimen identification relevance by MALDI-TOF MS. It is interesting to note that lower LSVs were obtained for body parts from specimens collected in the field compared to the laboratory reared ones, and this phenomenon was less pronounced for the idiosomes than for the legs. Solely MS spectra from laboratory reared specimens were included in the database, which could explain higher matching level for this group (Fig 6). Nevertheless, correct relevant identifications were also obtained for field specimens of both body parts. Despite the proximity of MS spectra between the legs and capitula of *I*. *ricinus*, for specimen identification by MS spectra query against the MS database, the use of the same body part, homogenized in the conditions as those included in the MS database, is recommended to improve matching level and then the reliability of identification.

## Conclusion

The present study failed to attribute specific MS profiles distinguishing *Borrelia-*infected and pathogen-free specimens for each body part tested. The low *Borrelia* inoculum and the absence of bacteria dissemination in the tick, at a distance from blood feeding, is likely explain this failure. Then, MALDI-TOF MS did not appear sufficiently noticeable for dual detection of *Borrelia* and tick species. The selection of a more specific body part such as gut and/or the use of other mass spectrometry strategies (i.e. targeted proteomics like Selected Reaction Monitoring in tandem with mass spectrometry [39]) could improve concomitant tick identification and/or pathogen detection [40]. However, the great reproducibility of the MS spectra generated from the three compartments would allow an identification using each one of these compartments. Moreover, the tick MS spectra specificity, from legs and idiosomes, opens new opportunities for the arthropod identification validation. Finally, the MALDI-TOF MS remains a rapid, economic, relevant and now worth validating tool for ticks and probably for arthropod identification.

## Abbreviations

sl: sensu lato
ss: sensu stricto
MALDI-TOF MS: Matrix Assisted Laser Desorption/Ionization Time-of-Flight Mass Spectrometry
BSK: Barbour-Stoenner-Kelly-based media
PCR: Polymerase Chain Reaction
CCI: Composite Correlation Index
PCA: Principal Component Analysis
GA: Genetic algorithm
KNN: K Nearest Neighbourg
RC: recognition capability
CV: Cross Validation
LSV: Log Score Value

## References

[1] Stanek G, Wormser GP, Gray J, Strle F. Lyme borreliosis. Lancet Lond Engl. 2012;379: 461–473. 10.1016/S0140-6736(11)60103-7 21903253

[2] Cutler SJ, Ruzic-Sabljic E, Potkonjak A. Emerging borreliae—Expanding beyond Lyme borreliosis. Mol Cell Probes. 2016; 22–27. doi: 10.1016/j.mcp.2016.08.003 27523487 10.1016/j.mcp.2016.08.003

[3] Ferquel E, Garnier M, Marie J, Bernede-Bauduin C, Baranton G, Perez-Eid C, et al Prevalence of *Borrelia burgdorferi* sensu lato and *Anaplasmataceae* members in *Ixodes ricinus* ticks in Alsace, a focus of Lyme Borreliosis endemicity in France. Appl Environ Microbiol. 2006;72: 3074–3078. doi: 10.1128/AEM.72.4.3074-3078.2006 16598024 10.1128/AEM.72.4.3074-3078.2006 PMC1449083

[4] Yssouf A, Almeras L, Raoult D, Parola P. Emerging tools for identification of arthropod vectors. Future Microbiol. 2016;11: 549–566. doi: 10.2217/fmb.16.5 27070074 10.2217/fmb.16.5

[5] Karger A, Kampen H, Bettin B, Dautel H, Ziller M, Hoffmann B, et al Species determination and characterization of developmental stages of ticks by whole-animal matrix-assisted laser desorption/ionization mass spectrometry. Ticks Tick-Borne Dis. 2012;3: 78–89. doi: 10.1016/j.ttbdis.2011.11.002 22487425 10.1016/j.ttbdis.2011.11.002

[6] Yssouf A, Flaudrops C, Drali R, Kernif T, Socolovschi C, Berenger J-M, et al Matrix-assisted laser desorption ionization-time of flight mass spectrometry for rapid identification of tick vectors. J Clin Microbiol. 2013;51: 522–8. doi: 10.1128/JCM.02665-12 23224087 10.1128/JCM.02665-12 PMC3553915

[7] Kumsa B, Laroche M, Almeras L, Mediannikov O, Raoult D, Parola P. Morphological, molecular and MALDI-TOF mass spectrometry identification of ixodid tick species collected in Oromia, Ethiopia. Parasitol Res. 2016;115: 4199–4210. doi: 10.1007/s00436-016-5197-9 27469536 10.1007/s00436-016-5197-9

[8] Croxatto A, Prod’hom G, Greub G. Applications of MALDI-TOF mass spectrometry in clinical diagnostic microbiology. FEMS Microbiol Rev. 2012;36: 380–407. doi: 10.1111/j.1574-6976.2011.00298.x 22092265 10.1111/j.1574-6976.2011.00298.x

[9] Seng P, Drancourt M, Gouriet F, Scola BL, Fournier P-E, Rolain JM, et al Ongoing Revolution in Bacteriology: Routine Identification of Bacteria by Matrix-Assisted Laser Desorption Ionization Time-of-Flight Mass Spectrometry. Clin Infect Dis. 2009;49: 543–551. doi: 10.1086/600885 19583519 10.1086/600885

[10] Fotso Fotso A, Mediannikov O, Diatta G, Almeras L, Flaudrops C, Parola P, et al MALDI-TOF mass spectrometry detection of pathogens in vectors: the *Borrelia crocidurae*/*Ornithodoros sonrai* paradigm. PLoS Negl Trop Dis. 2014;8: e2984 doi: 10.1371/journal.pntd.0002984 25058611 10.1371/journal.pntd.0002984 PMC4109908

[11] Yssouf A, Almeras L, Terras J, Socolovschi C, Raoult D, Parola P. Detection of *Rickettsia* spp in ticks by MALDI-TOF MS. PLoS Negl Trop Dis. 2015;9: e0003473 doi: 10.1371/journal.pntd.0003473 25659152 10.1371/journal.pntd.0003473 PMC4319929

[12] Yssouf A, Almeras L, Berenger J-M, Laroche M, Raoult D, Parola P. Identification of tick species and disseminate pathogen using hemolymph by MALDI-TOF MS. Ticks Tick-Borne Dis. 2015;6: 579–586. doi: 10.1016/j.ttbdis.2015.04.013 26051210 10.1016/j.ttbdis.2015.04.013

[13] Tonetti N, Voordouw MJ, Durand J, Monnier S, Gern L. Genetic variation in transmission success of the Lyme borreliosis pathogen Borrelia afzelii. Ticks Tick-Borne Dis. 2015;6: 334–343. doi: 10.1016/j.ttbdis.2015.02.007 25748511 10.1016/j.ttbdis.2015.02.007

[14] Pérez-Eid C. Les tiques. Identification, biologie, importance médicale et vétérinaire Lavoisier 2007.

[15] Kern A, Collin E, Barthel C, Michel C, Jaulhac B, Boulanger N. Tick saliva represses innate immunity and cutaneous inflammation in a murine model of lyme disease. Vector Borne Zoonotic Dis. 2011;11: 1343–50. doi: 10.1089/vbz.2010.0197 21612525 10.1089/vbz.2010.0197

[16] Mbow ML, Rutti B, Brossard M. Infiltration of CD4+ CD8+ T cells, and expression of ICAM-1, Ia antigens, IL-1 alpha and TNF-alpha in the skin lesion of BALB/c mice undergoing repeated infestations with nymphal *Ixodes ricinus* ticks. Immunology. 1994;82: 596–602. 7835923 PMC1414904

[17] Kern A, Collin E, Barthel C, Michel C, Jaulhac B, Boulanger N. Tick saliva represses innate immunity and cutaneous inflammation in a murine model of Lyme disease. Vector Borne Zoonotic Dis Larchmt N. 2011;11: 1343–1350. doi: 10.1089/vbz.2010.0197 21612525 10.1089/vbz.2010.0197

[18] Guy EC, Stanek G. Detection of *Borrelia burgdorferi* in patients with Lyme disease by the polymerase chain reaction. J Clin Pathol. 1991;44: 610–611. 1856296 10.1136/jcp.44.7.610 PMC496808

[19] Rijpkema S, Golubić D, Molkenboer M, Verbeek-De Kruif N, Schellekens J. Identification of four genomic groups of *Borrelia burgdorferi* sensu lato in *Ixodes ricinus* ticks collected in a Lyme borreliosis endemic region of northern Croatia. Exp Appl Acarol. 1996;20: 23–30. 8746131 10.1007/BF00051474

[20] Duron O, Noël V, McCoy KD, Bonazzi M, Sidi-Boumedine K, Morel O, et al The Recent Evolution of a Maternally-Inherited Endosymbiont of Ticks Led to the Emergence of the Q Fever Pathogen, *Coxiella burnetii*. PLoS Pathog. 2015;11: e1004892 doi: 10.1371/journal.ppat.1004892 25978383 10.1371/journal.ppat.1004892 PMC4433120

[21] Hidri N, Barraud O, de Martino S, Garnier F, Paraf F, Martin C, et al Lyme endocarditis. Clin Microbiol Infect. 2012;18: E531–2. doi: 10.1111/1469-0691.12016 23043635 10.1111/1469-0691.12016

[22] Nebbak A, El Hamzaoui B, Berenger J-M, Bitam I, Raoult D, Almeras L, et al Comparative analysis of storage conditions and homogenization methods for tick and flea species for identification by MALDI-TOF MS. Med Vet Entomol. 2017; doi: 10.1111/mve.12250 28722283 10.1111/mve.12250

[23] Nebbak A, Willcox AC, Bitam I, Raoult D, Parola P, Almeras L. Standardization of sample homogenization for mosquito identification using an innovative proteomic tool based on protein profiling. Proteomics. 2016; doi: 10.1002/pmic.201600287 27862981 10.1002/pmic.201600287

[24] Diarra AZ, Almeras L, Laroche M, Berenger J-M, Koné AK, Bocoum Z, et al Molecular and MALDI-TOF identification of ticks and tick-associated bacteria in Mali. PLoS Negl Trop Dis. 2017;11: e0005762 doi: 10.1371/journal.pntd.0005762 28742123 10.1371/journal.pntd.0005762 PMC5542699

[25] Lafri I, Almeras L, Bitam I, Caputo A, Yssouf A, Forestier C-L, et al Identification of Algerian Field-Caught Phlebotomine Sand Fly Vectors by MALDI-TOF MS. PLoS Negl Trop Dis. 2016;10: e0004351 doi: 10.1371/journal.pntd.0004351 26771833 10.1371/journal.pntd.0004351 PMC4714931

[26] Stanek G, Wormser G, Gray J, Strle F. Lyme borreliosis. Lancet. Elsevier Ltd; 2012;379: 461–73. doi: 10.1016/S0140-6736(11)60103-7 21903253 10.1016/S0140-6736(11)60103-7

[27] Piesman J, Schneider BS, Zeidner NS. Use of quantitative PCR to measure density of *Borrelia burgdorferi* in the midgut and salivary glands of feeding tick vectors. J Clin Microbiol. 2001;39: 4145–4148. doi: 10.1128/JCM.39.11.4145-4148.2001 11682544 10.1128/JCM.39.11.4145-4148.2001 PMC88501

[28] Dunham-Ems SM, Caimano MJ, Pal U, Wolgemuth CW, Eggers CH, Balic A, et al Live imaging reveals a biphasic mode of dissemination of *Borrelia burgdorferi* within ticks. J Clin Invest. 2009;119: 3652–3665. doi: 10.1172/JCI39401 19920352 10.1172/JCI39401 PMC2786795

[29] Rudenko N, Golovchenko M, Edwards MJ, Grubhoffer L. Differential expression of *Ixodes ricinus* tick genes induced by blood feeding or *Borrelia burgdorferi* infection. J Med Entomol. 2005;42: 36–41. 15691006 10.1093/jmedent/42.1.36

[30] Rudenko N, Golovchenko M, Grubhoffer L. Gene organization of a novel defensin of *Ixodes ricinus*: first annotation of an intron/exon structure in a hard tick defensin gene and first evidence of the occurrence of two isoforms of one member of the arthropod defensin family. Insect Mol Biol. 2007;16: 501–507. doi: 10.1111/j.1365-2583.2007.00745.x 17651239 10.1111/j.1365-2583.2007.00745.x

[31] Dieme C, Yssouf A, Vega-Rúa A, Berenger J-M, Failloux A-B, Raoult D, et al Accurate identification of *Culicidae* at aquatic developmental stages by MALDI-TOF MS profiling. Parasit Vectors. 2014;7: 544 doi: 10.1186/s13071-014-0544-0 25442218 10.1186/s13071-014-0544-0 PMC4273427

[32] Jacquet M, Genné D, Belli A, Maluenda E, Sarr A, Voordouw MJ. The abundance of the Lyme disease pathogen *Borrelia afzelii*declines over time in the tick vector Ixodes ricinus. Parasit Vectors. 2017;10: 257 doi: 10.1186/s13071-017-2187-4 28545520 10.1186/s13071-017-2187-4 PMC5445446

[33] Beati L, Humair PF, Aeschlimann A, Raoult D. Identification of spotted fever group *rickettsiae* isolated from *Dermacentor marginatus* and Ixodes ricinus ticks collected in Switzerland. Am J Trop Med Hyg. 1994;51: 138–148. 7915498 10.4269/ajtmh.1994.51.138

[34] Van Treuren W, Ponnusamy L, Brinkerhoff RJ, Gonzalez A, Parobek CM, Juliano JJ, et al Variation in the Microbiota of *Ixodes* Ticks with Regard to Geography, Species, and Sex. Appl Environ Microbiol. 2015;81: 6200–6209. doi: 10.1128/AEM.01562-15 26150449 10.1128/AEM.01562-15 PMC4542252

[35] Uhlmann KR, Gibb S, Kalkhof S, Arroyo-Abad U, Schulz C, Hoffmann B, et al Species determination of Culicoides biting midges via peptide profiling using matrix-assisted laser desorption ionization mass spectrometry. Parasit Vectors. 2014;7: 392 doi: 10.1186/1756-3305-7-392 25152308 10.1186/1756-3305-7-392 PMC4158057

[36] Kahl O, Gern L, Eisen L, Lane RS. Ecological research on Borrelia burgdorferi sensu lato: terminology and some methodological pitfalls Lyme borreliosis: biology, epidemiology and control. CABI; First edition; 2002 pp. 29–46.

[37] Boyer PH, De Martino SJ, Hansmann Y, Zilliox L, Boulanger N, Jaulhac B. No evidence of *Borrelia mayonii* in an endemic area for Lyme borreliosis in France. Parasit Vectors. 2017;10: 282 doi: 10.1186/s13071-017-2212-7 28583197 10.1186/s13071-017-2212-7 PMC5460422

[38] Yssouf A, Parola P, Lindström A, Lilja T, L’Ambert G, Bondesson U, et al Identification of European mosquito species by MALDI-TOF MS. Parasitol Res. 2014;113: 2375–2378. doi: 10.1007/s00436-014-3876-y 24737398 10.1007/s00436-014-3876-y

[39] Schnell G, Boeuf A, Westermann B, Jaulhac B, Lipsker D, Carapito C, et al Discovery and targeted proteomics on cutaneous biopsies infected by borrelia to investigate lyme disease. Mol Cell Proteomics MCP. 2015;14: 1254–1264. doi: 10.1074/mcp.M114.046540 25713121 10.1074/mcp.M114.046540 PMC4424397

[40] Schnell G, Boeuf A, Westermann B, Jaulhac B, Carapito C, Boulanger N, et al Discovery and targeted proteomics on cutaneous biopsies: a promising work toward an early diagnosis of Lyme disease. Mol Cell Proteomics. 2015;14: 1254–64. doi: 10.1074/mcp.M114.046540 25713121 10.1074/mcp.M114.046540 PMC4424397

